# Numerical Simulation of the Water Vapor Separation of a Moisture-Selective Hollow-Fiber Membrane for the Application in Wood Drying Processes

**DOI:** 10.3390/membranes11080593

**Published:** 2021-07-31

**Authors:** Nasim Alikhani, Douglas W. Bousfield, Jinwu Wang, Ling Li, Mehdi Tajvidi

**Affiliations:** 1 School of Forest Resources, University of Maine, Orono, ME 04469-5755, USA; nasim.alikhani@maine.edu (N.A.); mehdi.tajvidi@maine.edu (M.T.); 2 Department of Chemical and Biological Engineering, University of Maine, Orono, ME 04469-5737, USA; bousfld@maine.edu; 3 USDA Forest Service, Forest Products Laboratory, Madison, WI 53726-2398, USA; jinwu.wang@usda.gov

**Keywords:** air dehydration, FEA modeling, hollow fiber membrane, mass transfer coefficient, Sherwood number, water vapor concentration

## Abstract

In this study, a simplified two-dimensional axisymmetric finite element analysis (FEA) model was developed, using COMSOL Multiphysics^®^ software, to simulate the water vapor separation in a moisture-selective hollow-fiber membrane for the application of air dehumidification in wood drying processes. The membrane material was dense polydimethylsiloxane (PDMS). A single hollow fiber membrane was modelled. The mass and momentum transfer equations were simultaneously solved to compute the water vapor concentration profile in the single hollow fiber membrane. A water vapor removal experiment was conducted by using a lab-scale PDMS hollow fiber membrane module operated at constant temperature of 35 °C. Three operation parameters of air flow rate, vacuum pressure, and initial relative humidity (RH) were set at different levels. The final RH of dehydrated air was collected and converted to water vapor concentration to validate simulated results. The simulated results were fairly consistent with the experimental data. Both experimental and simulated results revealed that the water vapor removal efficiency of the membrane system was affected by air velocity and vacuum pressure. A high water vapor removal performance was achieved at a slow air velocity and high vacuum pressure. Subsequently, the correlation of Sherwood (Sh)–Reynolds (Re)–Schmidt (Sc) numbers of the PDMS membrane was established using the validated model, which is applicable at a constant temperature of 35 °C and vacuum pressure of 77.9 kPa. This study delivers an insight into the mass transport in the moisture-selective dense PDMS hollow fiber membrane-based air dehumidification process, with the aims of providing a useful reference to the scale-up design, process optimization and module development using hollow fiber membrane materials.

## 1. Introduction

Fast developing and cutting-edge membrane separation technology has been widely used in environmental remediation, food, chemical, and pharmaceutical industries. The membrane separation processes operate without heating and therefore use less energy than conventional thermal separation processes that involve a phase change process, such as distillation, sublimation or crystallization. Among a broad range of applications, dense membranes for vapor/gas separation (also called moisture-selective dense membranes) became popular in industrial separation applications since the serial production of commercial polymeric membranes was implemented in the 1980s [1]. Literature review reveals that many polymer-based membrane materials, such as polydimethylsiloxane (PDMS), poly ether-block-amide (PEBAX), sulfonated poly(ether ether ketone) (SPEEK), can be used for the removal of water vapor (dehumidification) from air or gas streams and therefore have been applied in certain air conditioning units to improve the energy saving of building systems [2,3,4].The advantages of moisture-selective membrane technology include less energy consumption due to no phase change of water involved, simplicity in maintenance and operation, high selectivity, ease of scale up, and low initial cost [5,6].

In our previous study, we explored the potential application of moisture-selective polydimethylsiloxane (PDMS) membrane in the steam-kiln wood drying process since a considerable amount of water vapor and thermal energy is stored in exhaust air [7]. Recycling and reusing such waste thermal energy would improve the energy efficiency of the kiln-drying process. One solution was to dehydrate the water vapor in the hot and humid exhaust air and redirect the dehydrated hot air into the kiln to transport water vapor evaporated from the wood. In the lab-scale experiment, a hollow-fiber membrane-based air dehumidification system was set up and tested. Compared with a plate-frame membrane module, the hollow-fiber membrane module was chosen due to its compact size and the extremely large surface area per unit volume of the membrane module [2,8]. A brief description of the system and its performance is described in the section of Materials and Methods.

This study aims to investigate the mass transfer in the hollow-fiber membrane using a numerical simulation method and establish the correlation of Sherwood number (Sh), Reynolds number (Re) and Schmidt number (Sc). This correlation is key information to assist engineers in designing the proper size of the hollow-fiber module and suitable operation parameters for different capacities of wood drying kilns in order to achieve the fast removal of water vapor from humid air [9,10,11,12,13]. Ideally, this can be accomplished with mathematical models established based on mass transfer to simulate the operating conditions at industrial scale coupled with a combination of well-designed lab-scale experiments that target a similar performance at industrial scale as observed at lab-scale.

Nowadays, the mathematical models can be solved using a numerical analysis technique/software, such as COMSOL Multiphysics^®^ software. The finite element analysis (FEA) based software has been widely used to create multiple physics based models to simulate the movement of various entities, such as mass, momentum, or energy through a medium, fluid or solid. The simulation of membrane separation processes (such as gas–gas, gas–liquid, liquid–solid, etc.) has been carried out by many researchers using the function of computational fluid dynamics (CFD) models [11,14,15,16,17]. However, only a couple of studies focused on the water vapor separation of polymeric-based hollow fiber membranes. One relevant study compared the water vapor concentration within a hollow fiber membrane modeled using a CFD model and a random walk approach [16]. In addition, the workload needed to drive the feed flow to estimate the energy consumption was calculated and discussed. The random walk approach showed results that were in good agreement with commercial CFD software and experimental data. The simulation outcome can be used to find the optimum working conditions for a hollow fiber membrane module. Another study employed a CFD model to simulate the pressure-driven water vapor separation in different hollow fiber composite membrane for air dehumidification [17]. The ultra-thin moisture selective dense layer of the composite membrane was modeled as a permeable barrier and its permeation was defined by the boundary conditions of the membrane domain. The velocity, pressure, and water vapor concentration profiles and mass transfer process in one single hollow fiber membrane were solved, verified and analyzed.

As an effective technique, we also used the FEA method to study the mass transfer in the moisture selective dense PDMS hollow fiber membrane for air dehumidification in the process of energy saving in wood drying. An air dehumidification experiment was conducted to collect the data of water vapor removal from the feed air stream, which was used for model validation. A two-dimensional axisymmetric FEA model was developed to simulate the mass transfer in one single hollow-fiber membrane. Then the modeling results of water vapor concentration at different feed air velocities were used to establish the correlation of Sh–Re–Sc. This correlation could be used for optimization and design of the hollow fiber membrane and to facilitate the industrial-scale application of PDMS hollow fiber membrane modules.

## 2. Materials and Methods

### 2.1. Materials

A polydimethylsiloxane (PDMS) membrane material was selected, which had a high water vapor permeability of 36,000 Barrer and an acceptable H_2_O/N_2_ selectivity of 129 [7,18]. A small hollow fiber PDMS membrane module enclosing bundles of hollow fibers was purchased from PermSelect-MedArray Inc., Ann Arbor, MI, USA. Table 1 lists the geometric information of the membrane module and single hollow fiber and the physical properties of PDMS membrane material, which were used as inputs in the FEA model.

### 2.2. Methods

#### Experimental Setup and Testing Procedure

**Figure 1:**
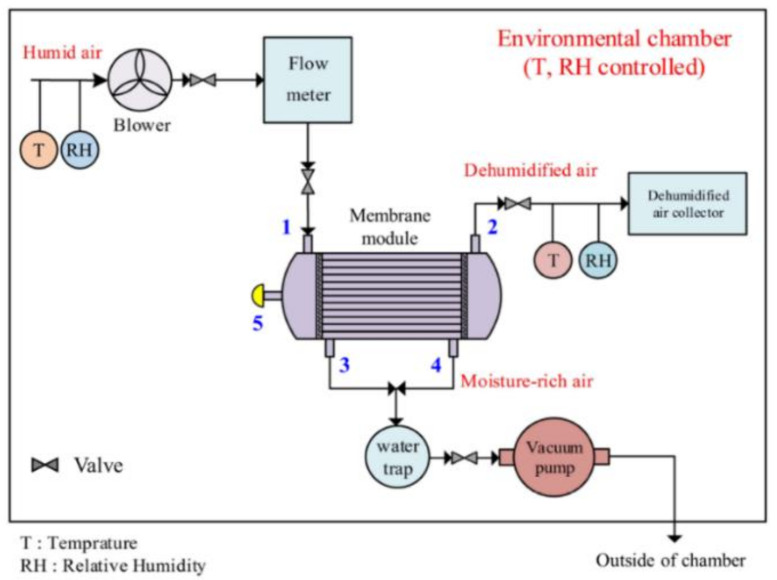
Schematic of a lab-scale membrane air dehumidification system. Reproduced with permission from Reference [7]. Copyright 2021 Wood and fiber Science.

As shown in Figure 1, the lab-scale membrane system was placed in a temperature- and RH-controlled environmental chamber. The humid air entered the membrane module through inlet 1. The air flow rate was controlled by an air blower and monitored by a mass/volume flow meter. The humid air passed through the lumen of hollow fibers and the dehydrated air came out of outlet 2, which was collected for the measurement of RH. The temperature and RH of both humid air and dehydrated air were monitored and recorded with time at an interval of 10 s by temperature and humidity sensors and a data logging system. The water vapor that permeated through the wall of hollow fibers came out of outlet 3 and outlet 4 and was expelled to the outside of the environmental chamber through a vacuum pump. The vacuum pump was equipped with a vacuum regulator and water vapor trap. Port 5 was blocked during the whole testing process. The RH values measured were then converted to water vapor concentrations in air.

The hollow fiber membrane system was operated at a constant temperature of 35 °C. The variable operation parameters are air flow rate, vacuum pressure, and initial RH. A total of nine (9) combinations were designed, which are listed in Table 2. Each combination was repeated three (3) times. The air flow rate and initial RH were converted to air velocity and concentration of water vapor, which are the required inputs in the FEA model.

The efficiency of water vapor removal of the air dehumidification membrane was calculated from Equation (1), which is used to compare the experimental results and modeling results [7].

(1) $$\mathrm{Efficiency}\left(\%\right)=\frac{\left(C_{w,\mathrm{in}}-C_{w,\mathrm{out}}\right)}{C_{w,\mathrm{in}}}\times 100$$

where, C_w,in_ (mol/m^3^) and C_w,out_ (mol/m^3^) are the initial and final concentrations of water vapor in air, respectively.

### 2.3. Finite Element Analysis Modeling

#### 2.3.1. Physical Model of the Hollow Fiber Membrane

**Figure 2:**
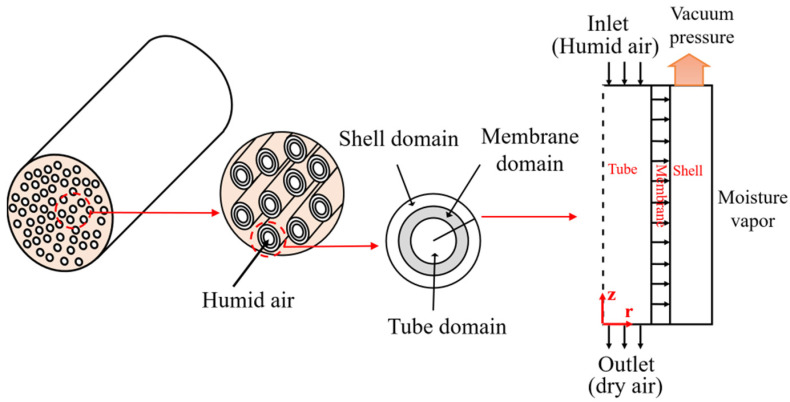
Schematic of a hollow fiber membrane module and the three domains of a hollow fiber membrane.

Figure 2 illustrates a schematic of a hollow fiber membrane module for air dehumidification and the three domains of tube, membrane, and shell, which are divided into one single hollow fiber membrane. In general, the air dehumidification process is accomplished by both convective and diffusive mass transfer, which is regarded as an isothermal process. Water vapor and air are regarded as ideal gases. As the humid air flows inside the tube (i.e., lumen) of the fiber, the air transports along the length of the fiber at a constant flow rate. Gas species diffuse in the tube along the fiber direction and in the membrane across the wall thickness of the fiber [25]. Diffusion in the membrane follows Fick’s first law. A well-known solution-diffusion mechanism is used to describe gas/vapor separation in dense membrane [1]. Gas molecules are first adsorbed to the surface of the inner wall of the membrane and then they diffuse in the membrane and migrate to the surface of the outer wall of the membrane. On the shell domain, the gas molecules are desorbed from the outer wall of the membrane by applying a vacuum pressure. The mass transport process is convection dominated in the tube and shell domains, and diffusion dominated in the membrane. The latter is not affected by the bulk flow of the air stream. Because of the higher solubility and diffusivity of water vapor molecules (H_2_O) in the membrane, H_2_O in humid air can transport across the membrane layer to the shell domain more quickly and easily than nitrogen (N_2_) and oxygen (O_2_) molecules at the same condition. Hence, a majority of N_2_ and O_2_ are retained in the tube domain to achieve the goal of air dehumidification.

Based on the assumption of the homogenous geometry and material of all hollow fiber membranes and a uniform distribution in the module, the simulation of water vapor transfer is simplified to model a single hollow fiber. A cylindrical coordinate system is used in the FEA model: z-axis is along the length direction of the fiber and r-axis is along the radius direction of the hollow fiber.

#### 2.3.2. Governing Equations of Mass Transfer

The convection mass transfer in a hollow fiber membrane can be described using a general continuity equation that complies with the law of mass conservation [16,26,27], in Equation (2).

(2) $$\frac{\partial C_{i}}{\partial t}=-\left(\nabla \cdot C_{i}V\right)-\left(\nabla \cdot J_{i}\right)+R_{i}$$

where, C_i_ (mol/m^3^), J_i_ (mol/(m^2^∙s)), V (m/s) and t (s) are concentration, diffusive flux, velocity and time, respectively. i denotes the gas species in air. R_i_ is the reaction rate of species i, which is zero because no chemical reaction is involved in the air dehumidification process [15,23,24].

In this study, water vapor is the target gas species, i.e., i = w is used in the following discussion.

Under the steady-state mass transfer, i.e., Fick’s first law, Equation (2) can be simplified as follows:

(3) $$V_{z}\frac{\partial c_{w}}{\partial z}=D_{w,j}\left[\frac{1}{r}\frac{\partial C_{w}}{\partial r}+\frac{\partial^{2\;}C_{w}}{\partial r^{2}}+\frac{\partial^{2\;}C_{w}}{\partial z^{2}}\;\right]$$

where, V_z_ (m/s) is the velocity of air in z direction. C_w_ (mol/m^3^) is the concentration of water vapor. D_w,j_ is the diffusion coefficient of water vapor in a substrate (j = a as in air; m as in membrane). r and z refer to the radial and axial coordinates, respectively, as shown in Figure 2.

The velocity distributions in the tube and shell domains are obtained by solving Navier–Stokes equation [25], Equation (4):

(4) $$\rho \frac{\partial \;\vec{V}}{\partial t}=-\nabla P+\mu \nabla^{2}\;\vec{V}+\rho g$$

where, ρ (kg/m^3^) is the density of air. V (m/s) is the velocity of air. P (kPa) is pressure. µ (kg/(m·s)) is the viscosity of air. g (m/s^2^) is the standard acceleration due to gravity.

Specially, the velocity distribution of air in the tube domain is regarded to follow the Newtonian laminar flow because the Re number calculated using the experimental data ranges from 0.32 to 0.53 (≤2300).

In the membrane domain, only water vapor diffusion governs the vapor transfer. Therefore, the velocity in the membrane domain is zero.

The boundary conditions applied in the hollow fiber system are described below:

- Tube side/domain
  The boundary conditions applied in the tube side are described below:
  (5) $$\mathrm{At}\;z=L,\;C_{w}=C_{w,\mathrm{in}}$$

Henry’s Law is applied to the interface of air (in the tube) and the inner wall surface of membrane [25,26,28]:

(6) $$\mathrm{At}\;r=r_{1},\;C_{w,m}=K\times C_{w,t}\;\left(\mathrm{No}\;\mathrm{slip}\;\mathrm{condition}\right)$$

where, K is a partition coefficient of water vapor in two phases of gas (i.e., air) and solid (i.e., membrane), which is determined by the solubility of water vapor in the gas and solid phases [29,30,31,32,33], Equation (7).

(7) $$\mathrm{logK}={\mathrm{logS}}_{w,\mathrm{PDMS}}-{\mathrm{logS}}_{w,\mathrm{air}}$$

where, S_w,a_ is the solubility of water vapor in air and S_w,PDMS_ is the solubility of water vapor in the PDMS membrane material used in this study.

(8) $$\mathrm{At}\;r=0,\;\frac{{\mathrm{dC}}_{w,t}}{\mathrm{dr}}=0\;(\mathrm{Symmetry})$$

- Membrane domain
  (9) $$\mathrm{At}\;r=r_{2},\;C_{w,m\;}=C_{w,s}\;(\mathrm{No}\;\mathrm{slip}\;\mathrm{condition})$$
- Shell domain
  (10) $$\mathrm{At}\;z=0,\;V_{z}=0,{\;C}_{w,s}=0$$
  (11) $$\mathrm{At}\;z=L,\;P=P_{\mathrm{Vacuum}}$$
  (12) $$\mathrm{At}\;r=r_{3},\;\frac{\partial C_{w,s}}{\partial r}=0\;(\mathrm{Symmetry}\;\mathrm{boundary};\;\mathrm{No}\;\mathrm{slip}\;\mathrm{condition})$$

#### 2.3.3. Geometry and Mesh Generation of FEA Model and Numerical Solution

**Figure 3:**
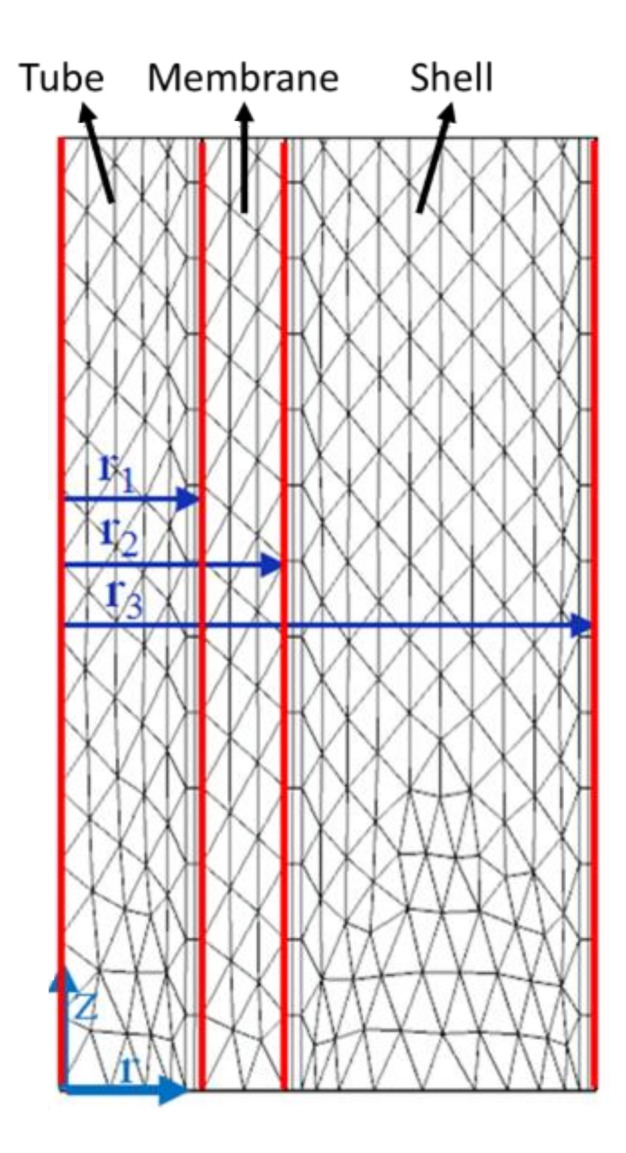
Schematic of a segment of the half longitudinal section of a single fiber mesh distribution.

An FEA model was developed via COMSOL software with a Computational Fluid Dynamics (CFD) module (COMSOL Multiphysics Version 5.4). As shown in Figure 3, a 2D-axisymmetric geometry was built to model the three domains of tube, membrane, and shell. The inner radius (r_1_) and outer radius (r_2_) of the tube are 95 µm and 150 µm, respectively. The radius of the shell domain (r_3_) is 350 µm, which was calculated using Happel’s free surface model, Equation (13) [25]. The total length (L) modeled is 0.1 m. The inlet of the tube domain for the humid air was set at z = L (i.e., $C_{w}=C_{w,\mathrm{in}}$), while the outlet of the tube domain for dehydrated air was set at z = 0. On the shell domain, the constant vacuum pressure was set at z = L (i.e., $P=P_{\mathrm{Vacuum}}$). A fine mesh size was chosen after doing a mesh convergence analysis and a total of about 70,000 mesh elements was created in the FEA model. Figure 3 shows a segment of the meshed geometry due to the extremely large length to radius ratio.

(13) $$r_{3}=r_{2}\times {\left(\frac{1}{1-\varphi }\right)}^{1/2}$$

where, φ is the volume fraction of the voids in the hollow fiber membrane module.

The simulation domains were solved by setting two physical modes in COMSOL software, namely, the laminar flow mode and the transport of vapor/gas species mode. The velocity field and the concentration field applied to the three domains were coupled and solved simultaneously. The static finite element analysis combined with error control was conducted with the PARDISO solver, which is a linear direct numerical solver. The convergence criteria were set to 10^−8^. As a result of simulation calculation, the concentration distribution of water vapor in the three domains was obtained.

### 2.4. Correlation of Sh–Re–Sc

The correlation relationship of Sh–Re–Sc numbers is established in the form of an exponential mathematical model [34], Equation (14).

(14) $$\mathrm{Sh}={\mathrm{ARe}}^{B}{\mathrm{Sc}}^{C}$$

where, A, B, and C are constants. Sh, Re and Sc numbers are calculated in Equations (15)–(17) [8,35].

(15) $$\mathrm{Sh}=\frac{k_{t}D}{D_{w,a}}$$

(16) Re=ρV¯Dμ

(17) $$\mathrm{Sc}=\frac{\mu }{\rho D}$$

The performance of a segment of the membrane for each condition can be used to obtain the overall mass transfer coefficient (k_o_) given in Equation (18) and derived in the Appendix A [8,25,36]. If the diffusion through the membrane is put in terms of a mass transfer coefficient of the membrane (k_m_) as in Equation (19), k_o_ is a sum of resistances given in the Appendix A. The shell side coefficient (k_s_) is found to be small compared to the others. Therefore, the overall and membrane coefficient (k_o_ and k_m_) can be used to calculate the tube side mass transfer coefficient (k_t_) for the particulate conditions in the FEA given in Equation (20).

(18) $$k_{o}=\frac{{\mathrm{DV}}_{z}}{4\;L}\ln\frac{C_{w,\mathrm{out}}}{C_{w,\mathrm{in}}}$$

(19) $$k_{m}=\frac{D_{w,m}}{T}$$

(20) $$k_{t}=\frac{k_{o}\times k_{m}}{k_{m}-k_{o}}$$

where, D (m) is the diameter of one fiber.

## 3. Results and Discussion

### 3.1. Experimental Results

The PDMS membrane system could quickly remove the water vapor from the air. It was observed that a significant drop of RH occurred in the first five minutes and then the RH remained constant at a lower value. The duration of each run was 30 min. The averaged RH value of dehydrated air calculated using the data collected in the last ten minutes was converted to the concentration of water vapor and used in the following discussion. A summary of the final concentrations of water vapor in dehydrated air (mean and standard derivation (SD) values) is provided in Table 3.

### 3.2. FEA Modeling Results

#### Water Vapor Concentration Profile in Three Domains

**Figure 4:**
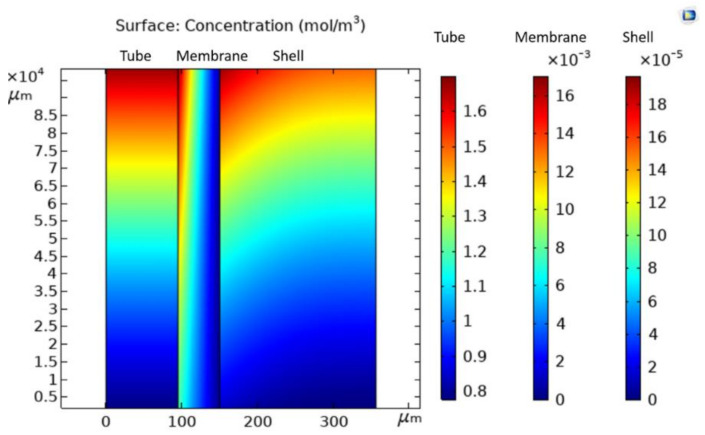
Distribution of concentration of water vapor in three domains of a hollow fiber membrane (air velocity = 0.037 m/s, initial concentration of water vapor = 1.72 mol/m^3^, and vacuum pressure = 77.9 kPa).

**Figure 5:**
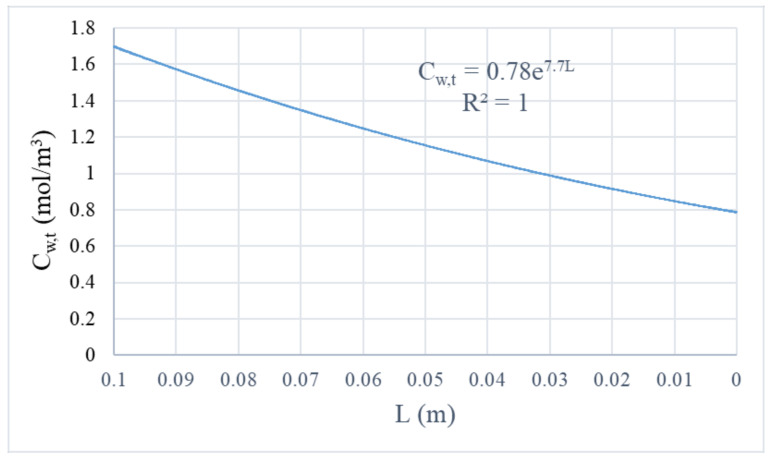
Axial concentration distribution of water vapor in the tube domain of hollow fiber membrane (air velocity = 0.028 m/s, initial concentration of water vapor = 1.72 mol/m^3^, and vacuum pressure = 67.7 kPa).

Figure 4 illustrates the concentration distribution of water vapor in a hollow fiber membrane under the operation condition No. 5 in Table 3. It is pointed out that, for demonstration only, the direction of fiber length was scaled down by 200X due to an extremely large ratio of a single fiber length and radius. In the tube domain, the humid air with an initial water vapor concentration of 1.72 mol/m^3^ flows from the top edge of the tube domain (at z = L), while dehydrated air with a final water vapor concentration of 0.79 mol/m^3^ flows out from the bottom edge of the tube domain (at z = 0). The decrease of water vapor concentration along the fiber length is plotted in Figure 5, which data was extracted by averaging the results of water vapor concentration at the same height of the fiber length direction in the tube domain. It reveals that the drop of water vapor concentration with the fiber length follows an exponentially decreasing trend. In the membrane domain, the concentration of water vapor varies from 0 to 0.016 mol/m^3^. Across the thickness of the membrane, the decrease in the concentration of water vapor is clearly observed. In the shell domain, the water vapor concentration is approximately zero, denoted by the scale bar in Figure 4. The water vapor concentration distribution is in line with the results discussed in other studies related to membrane separation [16,25].

### 3.3. Model Validation

**Figure 6:**
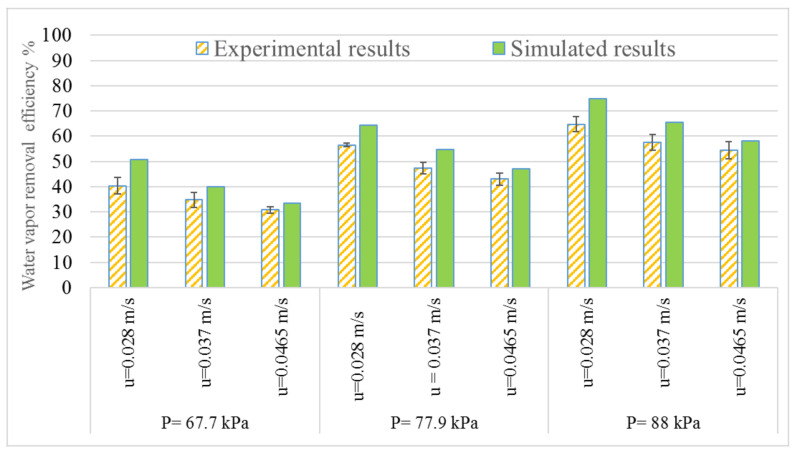
The concentration of water vapor of dehumidified air at outlet of PDMS membrane.

To validate the modeling results, the water vapor concentration of the dehydrated air was obtained by using an averaged value of water vapor concentration distributed along the bottom edge (at z = 0) of the tube domain. The averaged results are listed in Table 3. The data in Table 3 were used to calculate the efficiency of water vapor removal. Both the simulated and experimental results are plotted in Figure 6. Overall, the experimental results are lower than the modeling simulated results to different degrees. It is noticeable that the difference between the experimental results and simulated results is reduced with the increase of air velocity, regardless of vacuum pressure and initial water vapor concentration. This is acceptable since the FEA model was developed based on assumptions made for ideal situations and some parameters in the model were from reference articles. Meanwhile, slight fluctuations of temperature and RH were observed during the testing. Both the experimental results and simulated results show the same trend of the influence of two operation parameters, air velocity and vacuum pressure, in the water vapor removal efficiency. The best performance of water vapor removal efficiency was achieved at the lowest air velocity (i.e., 0.028 m/s) and highest vacuum pressure (i.e., 88 kPa) applied. Increasing air velocity from 0.028 m/s to 0.0465 m/s resulted in a slight decrease by approximately 25% in the efficiency at the lowest vacuum pressure of 67.7 kPa. As increasing the vacuum pressure to 88 kPa, the decrease in the efficiency due to the increase of air velocity was reduced to about 15%. A detailed discussion on the influence of operation parameters on the efficiency was given in our previous study [7]. The FEA model developed in this study is in good agreement with the experimental values for different values of air velocity and vacuum pressures.

### 3.4. Correlation Relationship of Sh–Re–Sc Numbers

**Figure 7:**
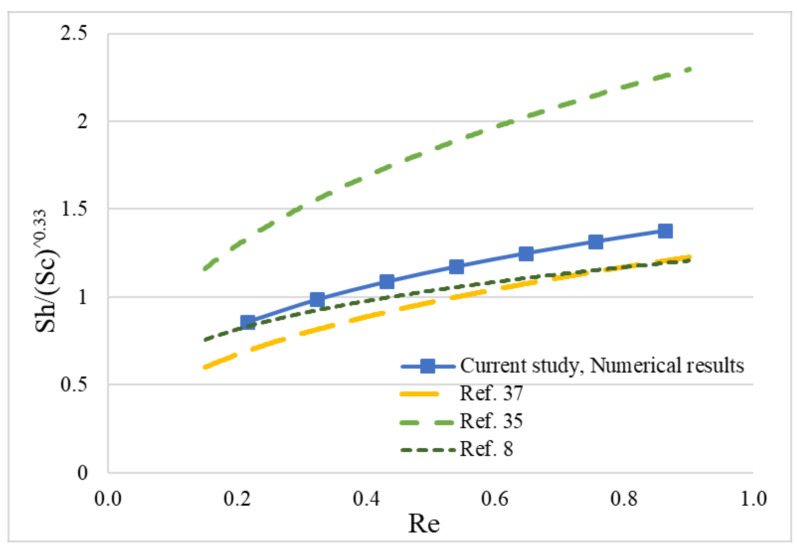
Correlation among Re, Sc and Sh numbers, based on both of the modeling results and experimental results of this study and the results of previous studies.

The verified FEA model was further used to assist in establishing a correlation relationship of Sh–Re–Sc numbers at a constant initial water vapor concentration of water vapor (i.e., 1.72 mol/m^3^), temperature (35 °C) and vacuum pressure (77.9 kPa) that is applied at the shell domain of the membrane system. These parameters were set as constant because the initial water vapor had little influence in the water vapor removal efficiency of the membrane and the temperature and vacuum pressure represented the medium level of operation conditions [7]. Only the inlet air velocity was set in a broad range of 0.019 m/s to 0.075 m/s at an interval of 0.009 m/s. The final water vapor concentration of dehydrated air was calculated using the FEA model. Equations (14)–(20) were used to establish the correlation of Sh–Re–Sc, in Figure 7. Among three constants of A, B, and C in Equation (14), A and B were obtained by doing a regression analysis. Constant C, the power of Sc number, was set as 0.33, which was determined based on literature that studied similar hollow fiber membrane modules [8,35,37].

Figure 7 illustrates the correlations of Sh–Re–Sc in other studies [8,35,37]. The two studies regarding the hollow-fiber membrane module system developed a 2-D FEA model to simulate the fluid flow and mass transfer in hollow fiber membrane systems [8,35]. The FEA modeling was used in the analysis of the correlation of Sh–Re–Sc. Another study regarding oxygen-liquid water separation of hollow fiber membrane module system discussed the mass transfer and the correlation of Sh–Re–Sc in terms of an experimental approach [37]. Although the FEA model developed in this study is a simplified model (one hollow fiber model), the simulated results are fairly acceptable, and the model can be used for membrane material screening and geometry optimization analysis. The correlation of Sh–Re–Sc numbers using the data set calculated by FEA model is presented in Equation (21). It can be seen in Figure 7 that the correlation obtained from the FEA model, is compatible with the correlations obtained from previous studies.

(21) Sh=1.45Re0.34Sc0.33

## 4. Conclusions

In this study, a simplified two-dimensional axisymmetric finite element analysis model was developed, using COMSOL Multiphysics^®^ software, to investigate the water vapor separation of a dense PDMS hollow fiber membrane module system for air dehumidification with potential applications in wood drying processes. In the FEA model, one single hollow-fiber was modelled. The convection mass transfer was calculated using a continuity equation and a momentum equation accompanied by well-defined boundary conditions. The water vapor concentration of dehydrated air obtained by solving the coupled equations using the finite element analysis model agreed well with the experimental data with a difference of less than 20%. The validated model was then used to calculate the mass transfer coefficient of water vapor and Sherwood number. The water vapor removal efficiency of the membrane system was affected by air velocity and vacuum pressure. A high water vapor removal performance was achieved at a slow air velocity and high vacuum pressure. A correlation of Sh–Re–Sc was also established using modeling results. The FEA model and relevant findings could be used in the design, process optimization and module development using hollow fiber membrane.

## Figures and Tables

**Table 1:** Geometric information of the hollow fiber membrane module and single hollow fiber and physical properties of PDMS membrane material.

Parameters	Value
Total surface area [19]	1 m^2^
Number of hollow fibers, n	12,600
Volume fraction, φ	0.887
Fiber inner radius, r_1_ [19]	95 µm
Fiber outer radius, r_2_ [19]	150 µm
Fiber wall thickness, T [19]	55 µm
Fiber length, L [19]	0.1 m
Diffusion coefficient of water vapor in PDMS membrane at 35 °C, D_w,m_ [20,21]	1.70 × 10^−8^ m^2^/s
Diffusion coefficient of water vapor in air at 35 °C, D_w,a_ [22]	2.67 × 10^−5^ m^2^/s
Solubility of water vapor in air at 35 °C, S_w,a_ [23]	0.036 g/g_air_
Solubility of water vapor in PDMS membrane at 35 °C, S_w,PDMS_ [24]	3.00 × 10^−4^ g/g_polymer_

**Table 2:** Experimental design of operating hollow-fiber membrane air dehumidification system.

ID	Operation Parameters			
	Air Velocity, m/s	Vacuum Pressure, kPa	Initial RH	Initial Concentration of Water Vapor, C_w,in_ (mol/m^3^)
1	0.028	67.7	75%	1.72
2	0.037	67.7	65%	1.48
3	0.0465	67.7	75%	1.72
4	0.028	77.9	65%	1.48
5	0.037	77.9	75%	1.72
6	0.0465	77.9	85%	1.96
7	0.028	88.0	75%	1.72
8	0.037	88.0	65%	1.48
9	0.0465	88.0	75%	1.72

**Table 3:** Summary of experimental and modeling results.

ID	Air Velocity, m/s	Vacuum Pressure, kPa	Initial Concentration of Water Vapor, C_w,in_ (mol/m^3^)	Final Concentration C_w,out_ (mol/m^3^)	
				Experimental Results	FEA Modeling Results
1	0.028	67.7	1.72	0.88 ± 0.03	0.73
2	0.037	67.7	1.48	1.12 ± 0.03	1.03
3	0.0465	67.7	1.72	1.19 ± 0.01	1.14
4	0.028	77.9	1.48	0.64 ± 0.01	0.53
5	0.037	77.9	1.72	0.91 ± 0.02	0.79
6	0.0465	77.9	1.96	0.98 ± 0.02	0.91
7	0.028	88.0	1.72	0.52 ± 0.03	0.37
8	0.037	88.0	1.48	0.73 ± 0.03	0.59
9	0.0465	88.0	1.72	0.78 ± 0.04	0.72

**Figure A1:**
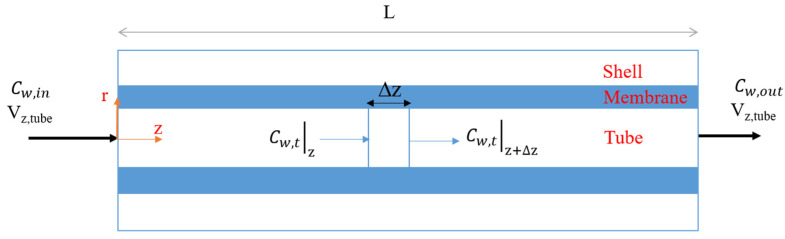
Schematic of a single hollow fiber.

## Data Availability

Not applicable.

## Abbreviation

Nomenclature:	
A	constant parameter
B	constant parameter
C	constant parameter
C_w,in_	concentration of water vapor in the inlet air
C_w,out_	concentration of water vapor in the outlet air
C_w,t_ (mol/m^3^)	concentration of water vapor in tube
C_w_ (mol/m^3^)	concentration of water vapor
C_w,s_ (mol/m^3^)	concentration of water vapor in shell
C_w,m_ (mol/m^3^)	concentration of water vapor in membrane
C_i_ (mol/m^3^)	concentration of i species
C_w_ (mol/m^3^)	concentration of water vapor
D (m)	diameter of one fiber
D_w,j_	diffusion coefficient of water vapor in a substrate
D_w,a_ (m^2^/s)	diffusion coefficient of water vapor in air
D_w,m_ (m^2^/s)	diffusion coefficient of water vapor in membrane
g (m/s^2^)	standard acceleration due to gravity
i	denotes the gas species in air
J_i_ (mol/m^2^·s)	diffusive flux
k	mass transfer coefficient
K	partition coefficient of water vapor in two phases
k_m_ (m/s)	membrane side mass transfer coefficient
k_o_ (m/s)	overall mass transfer coefficient
k_s_ (m/s)	shell side mass transfer coefficient
k_t_ (m/s)	tube side mass transfer coefficient
L (m)	fiber length
n	number of hollow fibers,
P (kPa)	pressure
P_vacuum_ (kPa)	vacuum pressure
R	radial direction
r_1_ (µm)	inner radius of the fiber
r_2_ (µm)	outer radius of the fiber
r_3_ (µm)	radius of the shell
Re	Reynolds number
RH	relative humidity
R_i_	reaction rate of species i
Sc	Schmidt number
Sh	Sherwood number
S_w,air_	solubility of water vapor in air
S_w,PDMS_	solubility of water vapor in PDMS
T (µm)	thickness of the membrane.
t (s)	time
$\bar{V}$	average velocity of the fluid inside the tube
V (m/s)	velocity
V_z_ (m/s)	velocity of air in z direction
V_z,shell_ (m/s)	velocity of air in the shell in z direction
V_z,tube_ (m/s)	velocity of air in the tube in z direction
w	water vapor
Z	axial direction
Greeks:	
ρ (kg/m^3^)	density of air
µ (kg/(m.s))	viscosity of air
φ	volume fraction

## Appendix A

In this section, a mass balance equation is described to calculate the overall mass transfer coefficient, k_o_, and the mass transfer coefficients in tube (k_t_), membrane (k_m_), and shell (k_s_) domains.

Taking a full-length hollow fiber membrane as a model, a differential equation of mass balance in terms of water vapor concentration and average air velocity is established in Equation (A1). The membrane is also divided into three domains: tube, membrane, and shell domains. A cylindrical ordination system is used, Figure A1.

(A1) $${\left.C_{w,t}\right|}_{z\;}V_{z}\frac{{\pi D}^{2}}{4}-{\left.C_{w,t}\right|}_{z+\Delta z\;}V_{z}\frac{{\pi D}^{2}}{4}-k_{o}\;\left({\left.C_{w,t}\right|}_{z\;}-{\;C}_{w,s}\right)\;\pi D\Delta z=0.$$

where, $C_{w,t}$ (mol/m^3^) is concentration of water vapor at a differential length, Δz, in the tube domain. $C_{w,s}$ (mol/m^3^) is the concentration of water vapor in the tube domain. $V_{z}$ (m/s) is the velocity of humid air following into the tube domain. D (m) is the diameter of a single hollow fiber. $k_{o}$ (m/s) is the overall mass transfer coefficient of water vapor.

It is assumed that mass transfer in r direction is constant. In our study, $C_{w,s}$ was unneglectable because the water vapor molecules migrated to the shell domain were immediately removed by a vacuum pump applied. In Equation (A1), it is treated as zero, then Equation (A1) can be further rewritten as Equation (A2):

(A2) $$k_{o}=\frac{{\mathrm{DV}}_{z}}{4\;L}\ln\frac{C_{w,\mathrm{out}}}{C_{w,\mathrm{in}}}$$

k_o_ comprises three components: k_t_, k_m_, k_s_ [10,31]. The relationship among them is given in Equation (A3):

(A3) $$\frac{1}{k_{o}}=\frac{1}{k_{t}}+\frac{1}{k_{m}}+\frac{1}{k_{s}}$$

k_m_, is given in Equation (A4) [8]:

(A4) $$k_{m}=\frac{D_{w,\;m}}{T}$$

where, D_w,m_ (m^2^/s) is the diffusion coefficient of water vapor in the membrane, T (µm) is the thickness of the membrane.

The water vapor molecules migrated to the shell domain were removed rapidly by a vacuum pump. Therefore, k_s_ is redeemed as infinity, i.e., k_s_ = ∞. Correspondingly, the resistance of the shell domain is neglectable.

Combining Equations (A3) and (A4), k_t_ is obtained by using Equation (A5):

(A5) $$k_{t}=\frac{k_{o}\times k_{m}}{k_{m}-k_{o}}$$
